# Motor vehicle accident mortality by elderly drivers in the super-aging era: A nationwide hospital-based registry in Japan

**DOI:** 10.1097/MD.0000000000012350

**Published:** 2018-09-21

**Authors:** Tasuku Matsuyama, Tetsuhisa Kitamura, Yusuke Katayama, Tomoya Hirose, Takeyuki Kiguchi, Junya Sado, Kosuke Kiyohara, Junichi Izawa, Nobunaga Okada, Kotaro Takebe, Makoto Watanabe, Yuki Miyamoto, Yoshihiro Yamahata, Bon Ohta

**Affiliations:** a Department of Emergency Medicine, Kyoto Prefectural University of Medicine, Kyoto; b Division of Environmental Medicine and Population Sciences, Department of Social and Environmental Medicine, Osaka University Graduate School of Medicine, Suita; c Department of Traumatology and Acute Critical Medicine; d Emergency and Critical Care Center, Osaka Police Hospital, Osaka; e Kyoto University Health Services, Kyoto; f Department of Public Health, Tokyo Women's Medical University; g Intensive Care Unit, Department of Anesthesiology, The Jikei University School of Medicine, Tokyo, Japan.

**Keywords:** elderly drivers, mortality, motor vehicle accidents, prevalence

## Abstract

Motor vehicle accidents (MVAs) are one of the major public health burdens worldwide. In particular, MVAs by elderly drivers have been significantly increasing in recent years in industrialized countries. This study aimed to assess the MVA characteristics and outcomes caused by elderly drivers in Japan.

Japan Trauma Data Bank (JTDB) is a prospective, nationwide, hospital-based registry for trauma patients from 256 institutions in Japan. This study enrolled all MVA drivers older than the legal age for driving between 2004 and 2015. The included patients were divided into the following 3 groups: adults (aged ≤64 years), young-old (aged 65–74 years), and old-old (aged ≥75 years). The primary outcome was in-hospital mortality. The trend in the proportion of MVAs caused by the young-old or the old-old group was evaluated using the Cochran–Armitage trend test. To assess the association of the old-old group with in-hospital mortality, compared with the adult group, we used multivariable logistic regression analysis.

During the study period, a total of 236,698 trauma patients were registered, and 39,691 patients (16.8%) were eligible for our analysis. The proportion of MVAs caused by elderly drivers aged ≥65 years significantly increased from 11.7% in 2004 to 23.8% in 2015 (*P* < .001). As for the primary outcome, unadjusted in-hospital mortality increased with age, but decreased year-by-year irrespective of the age group. In multivariable logistic regression analysis, in-hospital mortality was significantly higher in the old-old group than in the adult group [17.3% (584/3372) vs 8.0% (2556/31,985); adjusted odds ratio 4.80; 95% confidence interval 4.06–5.67].

In the super-aging society of Japan, the proportion of MVAs by elderly drivers increased year-by-year, and the mortality rate was highest in those aged above 75 years.

## Abstract 2

Supplemental Digital Content is available in the text

## 1 Introduction

Motor vehicle accidents (MVAs) are one of the most important global public health problems. According to the World Health Organization, there are over 3400 deaths each day, and over 20 million nonfatal injuries every year on roads worldwide.^[1]^ However, in the recent decade, the number of MVA deaths has been decreasing among high-income countries. This is likely due to the active prevention and intervention strategy for MVAs.^[1,2]^

Many industrialized countries face the problem of population aging. In Japan, the proportion of elderly licensed drivers aged ≥65 years has been increasing, and in particular, the number of those aged 75 years has been twice as large in the past 10 years.^[3]^ In the United States, the number of licensed drivers aged ≥65 years in 2015 has been increasing by 1.5 times, compared with that in 1999.^[4]^ Many European countries are also experiencing a similar increasing trend.^[5]^ As a result, contrary to the statistics of all age groups, the number of MVAs caused by elderly drivers aged ≥65 years has increased.^[6]^ Importantly, the aging population in Japan will accelerate in the near future,^[7]^ and MVAs by elderly drivers will, therefore, pose a heavier burden on Japan, as well as other industrialized countries. Despite the urgent need for public health achievements in the reduction of MVAs by elderly drivers, there are no clinical studies investigating their actual impact with consideration of factors aside from patient age, such as the influence of sex, severity of trauma, and pre-existing medical conditions.

The Japanese Trauma Data Bank (JTDB) is a prospective, nationwide, hospital-based trauma registry in Japan, and ongoingly collecting data using uniform predefined dataform to assess the association between various factors and outcome. This registry was initiated in 2003, and approximately 230,000 trauma patients were registered by 2015.^[8]^ Using this database, the study aims to perform a detailed analysis of the trend, characteristics, and outcomes of MVAs caused by elderly drivers.

## 2 Methods

### 2.1 Study design, population, and setting

This study was a retrospective analysis of the JTDB database. The study period was from January 2004 to December 2015. The inclusion criteria were all MVA drivers with age older than the legal age for driving (≥18 years) among those who were transported to the participating hospital and were registered in the database. The exclusion criteria included those whose age or the outcome (death or survival) was unknown.

### 2.2 Japanese Trauma Data Bank

The JTDB was established in 2003, and authorized by the Japanese Association for the Surgery and Trauma (Trauma Surgery Committee) as well as the Japanese Association for Acute Medicine (Committee for Clinical Care Evaluation),^[9,10]^ similar to trauma databases in North America, Europe, and Oceania.^[11]^ During the study period, the number of institutions participating in JTDB increased from 55 in 2004 to 256 in 2015 (Supplementary Table).^[10]^ Data were collected through an online portal from participating institutions. In most cases, data registration was performed by the physicians and medical assistants who completed the abbreviated injury scale (AIS) coding course.^[11]^

In this registry, data were collected on trauma-related factors, including age, sex, mechanism of injury, pre-existing medical conditions, AIS code (version 1998), injury severity score (ISS), vital signs on hospital arrival, date and time courses from hospital arrival to discharge, medical treatments such as advanced airway management, red blood cell transfusion within 24 hours, and emergency surgical operation, as well as mortality at discharge.^[8]^ ISS was calculated from the top 3 scores of AIS in 9 sites classified by AIS code.

From the JTDB database, we extracted age, sex, pre-existing medical conditions, time of the day, day of the week, vital signs on hospital arrival, positive focused assessment with sonography for trauma (FAST) examination, maximum AIS score for each site, ISS, in-hospital treatment process, and outcome data. In this study, systolic blood pressure and Glasgow Coma Scale (GCS) were classified into several categories based on the Revised Trauma Score (RTS) definition. This study defined daytime as 9:00 to 17:59, and nighttime as 18:00 to 8:59.

### 2.3 Endpoints

The primary outcome of this study was in-hospital mortality, and the secondary outcome was the length of hospital stay (LOS) as well as the discharge location [home or not (other health care facility or other facility)] among survivors.

### 2.4 Statistical analysis

Included patients were divided into the following 3 age groups; adults aged ≤64 years, young-old aged 65 to 74 years, and old-old aged ≥75 years. We examined the normality to use the Kolmogorov–Smirnov test because of large sample size of this study and observed that all the continuous variables were not normally distributed. Therefore, the differences between the 3 groups were assessed using Chi-square test for categorical variables, and Kruskal–Wallis test for continuous ones. The trend in the proportion of MVAs caused by the young-old or the old-old group was evaluated using the Cochran–Armitage trend test. As for the primary end-point, we visually described the nonlinear relationship between the patients’ age and the estimated probability of in-hospital mortality using restricted cubic splines in the univariable logistic regression model for every 4-year stratum. In addition, we performed the join-point regression analysis to assess the 1-year trend in the MVA mortality. To assess the impact of the age category on in-hospital deaths, multivariable logistic regression model was applied with odds ratio and 95% confidence interval (95% CI) as the effect variables. The potential factors were gender, the number of preexisting medical condition category (0, 1, ≥2), calendar year, time of day (daytime/nighttime), day of the week (weekday/weekend and holiday), systolic blood pressure category on hospital arrival, GCS category on hospital arrival, and ISS category. Also, as for secondary outcomes, we performed a multiple regression analysis (LOS as dependent variable) and multivariable logistic regression (discharge location as dependent variable) adjusting for the same covariates listed above. All tests were 2-tailed, and a *P* value of <.05 was considered statistically significant. Statistical analysis was performed by SPSS version 22.0J (IBM Corp., Armonk, NY), and EZR (Saitama Medical Center, Jichi Medical University, ver. 1.32), which is a graphical user interface for R.^[12]^

### 2.5 Ethics

This study was approved by the ethics committee of Kyoto Prefectural University of Medicine. Personal identifiers were already removed from the JTDB database, and the requirement for informed consent was waived.

## 3 Results

**Figure 1:**
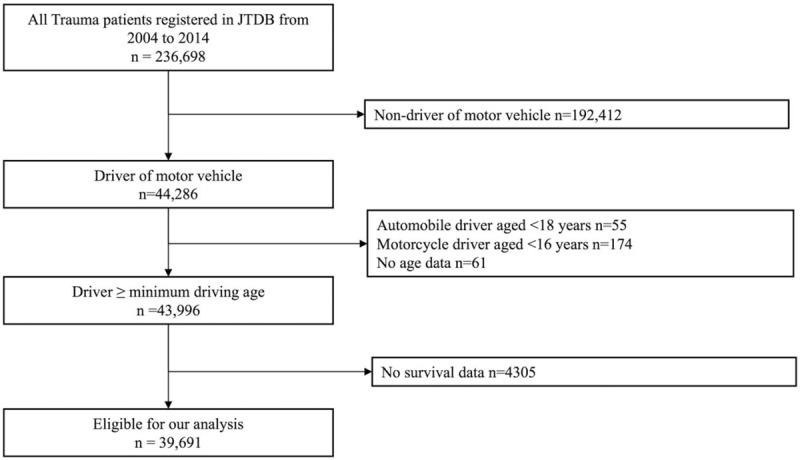
Flowchart of patient enrolment. JTDB = Japan Trauma Data Bank.

During the study period from 2004 to 2015, a total of 236,698 trauma patients were registered in JTDB. Excluding 192,412 nondrivers of motor vehicles, 290 MVA drivers aged younger than the legal age for driving or an unknown age, and 4305 patients with no survival data, 39,691 patients were included in our final analysis (Fig. 1).

**Figure 2:**
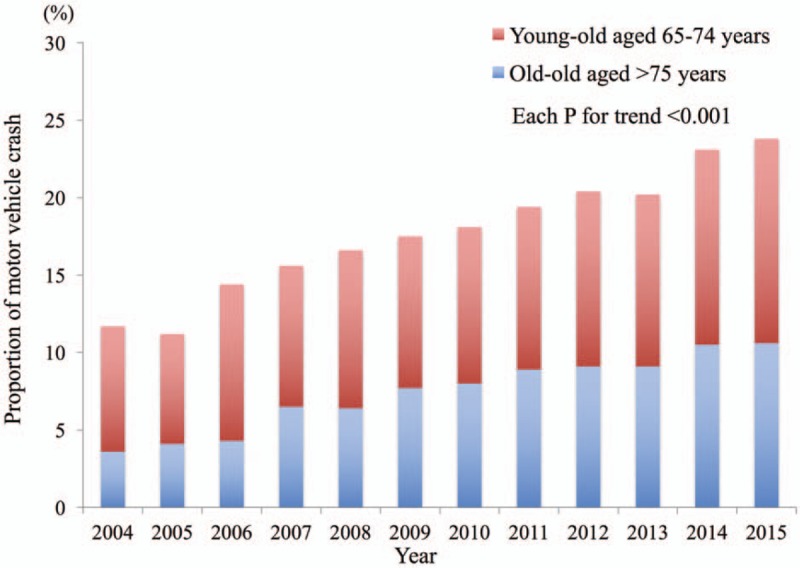
Prevalence of motor vehicle accidents caused by elderly drivers during the study period.

The proportion of MVAs caused by the young-old and the old-old group significantly increased from 8.1% and 3.6% in 2004 to 10.6% and 13.2% in 2015, respectively (*P* < .001) (Fig. 2). Patient characteristics and in-hospital treatment by age category are summarized in Table 1. The number of pre-existing medical conditions were higher in the elderly group. In addition, trauma severity, such as vital signs on hospital arrival, ISS, and the proportion of positive FAST examination, was higher in the elderly group. Elderly patients were more likely to receive some of the aggressive treatments such as advanced airway management and Red blood cell transfusion (RBC), but were less likely to receive emergency operation.

**Table 1:**
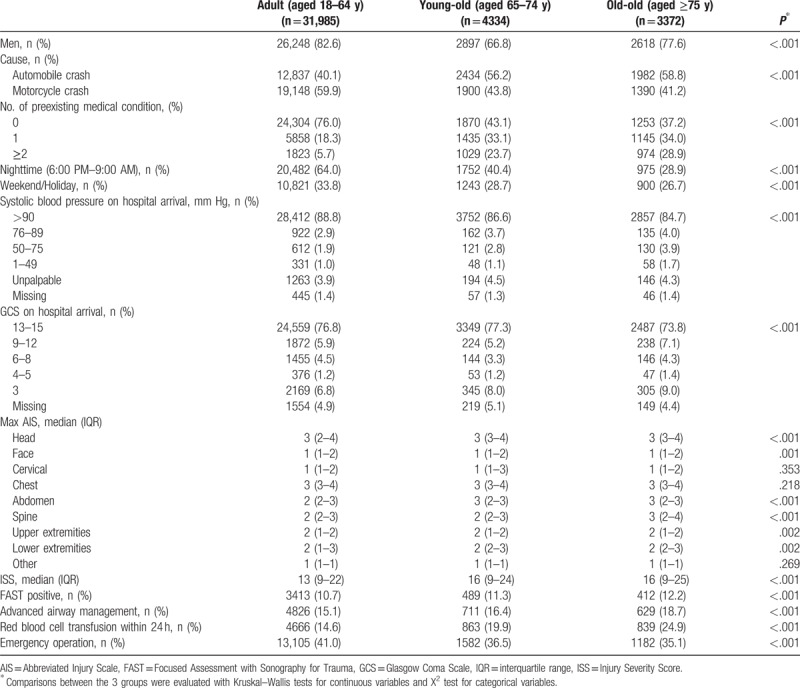
Patient characteristics and in-hospital treatments by age category.

**Figure 3:**
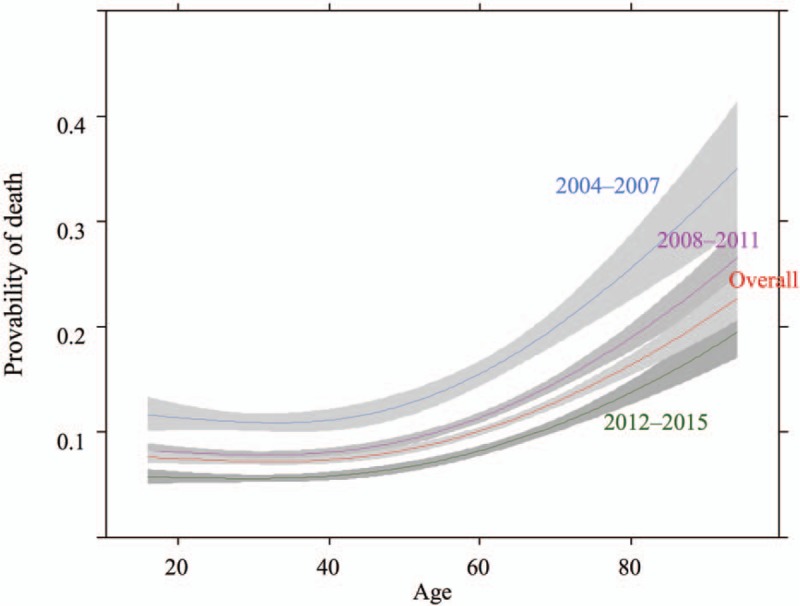
Probability of in-hospital death by patient's age per 4-year strata.

As for the primary outcome, in-hospital mortality increased with age, but decreased year-by-year irrespective of age (Fig. 3). During the study period, annual in-hospital mortality in all study patients, those aged 18 to 74 years, and aged ≥75 years decreased from 15.4%, 15.4%, and 17.6% in 2004 to 6.9%, 6.1%, and 14.1% in 2015, respectively. The join-point regression analysis demonstrated that the annual reduction in in-hospital mortality was 7.4%, 8.1%, and 7.4%, in all study patients, those aged 18 to 74 years, and aged ≥75 years, respectively, and no join-point was observed in each group during the study period. The proportion of in-hospital death was significantly higher in the old-old group than in the adult group [17.3% (584/3372) vs 8.0% (2556/31,985)] (Table 2). The multivariable logistic regression analysis also demonstrated that in-hospital mortality was significantly higher in the old-old group than in the adult group (adjusted odds ratio 4.80; 95% CI 4.06–5.67) (Table 2).

**Table 2:**
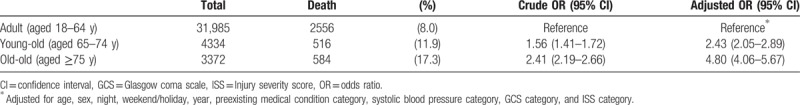
Death from motor vehicle accidents by age category.

In addition, even among survival cases, the old-old group was less likely to be discharged to home than the adult group [46.9% (1580/3372) vs 33.0% (10,553/31,985), odds ratio 0.42; 95% CI 0.39–0.46] and LOS was longer in the old-old group than in the adult group [median (interquartile range), 19 (8–41) vs 13 (4–31), *P* < .001]. These secondary outcomes showed the trend after adjusting for the important covariates (Tables 3 and 4).

**Table 3:**
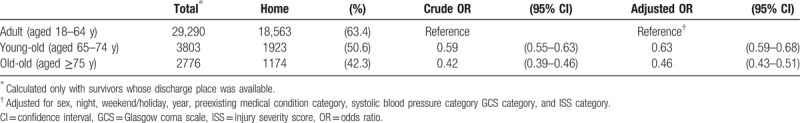
Discharge to home after motor vehicle accidents by age category among survivors.

**Table 4:**
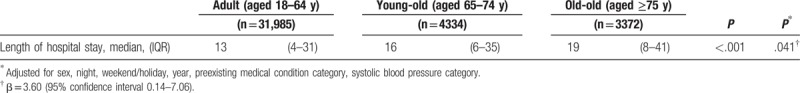
Length of hospital stay after motor vehicle accidents by age category among survivors.

## 4 Discussion

Using the nationwide trauma registry in Japan, we demonstrated that the proportion of MVAs caused by elderly drivers significantly increased during the study period. Further, although in-hospital mortality has improved year-by-year regardless of patient's age, even after adjusting for the potential prognostic factors, in-hospital mortality increased with age. In the super-aging society of Japan, this large-scale trauma registry enabled us to evaluate the prevalence and outcome of MVAs by elderly drivers. Our findings from the rapidly aging country may yield insights for better prevention and intervention strategies for MVAs caused by elderly drivers in Japan, as well as other industrialized countries.

In this study, the proportion of MVAs caused by elderly drivers showed an increasing trend during the study period. The aging population is progressing in many industrialized countries, and in the Global Age Watch Index 2015 overall ranking, the top ranking 20 were mostly occupied by industrialized countries, including the United States, European countries, as well as Japan.^[13]^ Further, MVAs caused by elderly drivers were one of the greater public health issues.^[4–6,14]^ For example, the number of elderly licensed drivers has been greater in recent decades, and there are a reported 19 elderly people killed and 712 injured in MVAs daily, on average.^[4]^ The proportion of elderly people, particularly those aged 75 years, is estimated to grow at an unprecedented rate, from 7.1% in 2000 to 18.1% in 2025, in Japan, outweighing all other countries.^[7]^ Therefore, the impact of Japan's aging population may be a potential indicator for future perspective of other countries.

The incidence and outcomes of MVAs by elderly drivers were influenced to a large extent by 2 factors: functional limitations and physical vulnerability. Functional limitations of elderly drivers, such as the decrease in visual and auditory acuity, judgment ability, and driving technique, increase the risk of MVA incidence. Although this study did not capture the direct cause for MVAs, according to the Metropolitan Police department of Japan, general reasons for MVAs caused by elderly drivers were as follows: “delay of the perception of the risk,” “misjudgment,” and “handling mistakes.”^[15]^ In general, elderly drivers are more likely to utilize the existing protective factors, such as seatbelt use, avoidance of driving at night, or impaired driving after drinking alcohol.^[16]^ Indeed, the proportion of MVAs at night was also lower in elderly populations. However, once the ability to drive decreases, improvement may be difficult to the same extent as that in youths. Instead, it might be more important to develop effective ways for the elderly to stay safer on the road, such as improving automatic driving technologies or making arrangements for alternative transportation.

Physical vulnerability of the elderly leads to worse prognosis after MVAs. The severity and mortality after MVAs becomes more serious under the influence of many age-related physical and physiological changes, such as muscle weakness, osteoporosis, or reduction in the function of all organ systems. In 1 study from the U.S., multiple frailty markers were more reliable factors of trauma prognosis than age itself.^[17]^ Patients’ past medical history and their regular medications may greatly affect the prognosis of trauma patients.^[10,18]^ For example, the mortality of trauma patients with the past medical history of congestive heart failure and taking β-blocker, or an anticoagulant medication, was reported to increase by 5 to 10 times.^[18]^ In addition, preinjury polypharmacy, which is now an important challenge facing elderly people, was found to be an independent prognostic factor for outcomes in trauma patients.^[19]^ Promoting regular exercise and avoiding unnecessary medication are beneficial for decreasing mortality of MVAs caused by elderly drivers.

This study observed that in-hospital mortality decreased year-by-year regardless of the patient's age. A prior study from the same database, which enrolled all trauma patients, demonstrated that the prognosis after trauma improved over time,^[20]^ and this improvement was likely multifactorial. One factor was due to the widespread use of standard trauma care across Japan through the educational course such as Japan Prehospital Trauma Evaluation and Care (JPTEC), Japan Advanced Trauma Evaluation and Care (JATEC), and Advanced Trauma Operative Management (ATOM) courses.^[21–23]^ Another factor may be partially explained by an improvement of the trauma care system. For example, 1 study from Japan observed that implementing a hybrid emergency room was associated with increased survival after potentially fatal trauma.^[24]^ Further, continued efforts to improve trauma care quality will be essential.

Japan has made great efforts to establish improved environments for elderly drivers. For example, the government of Japan implemented additional examinations for drivers aged ≥75 years in order to update their drivers’ licenses.^[25]^ Many western countries also take various measures for the driving safety of the elderly with dementia, such as close observation, discussions, and arranging alternative transportation.^[26,27]^ From a health care workers’ point of view, contributing to the development of an effective system would be important by means of assessing the driving ability of elderly driver arriving at a hospital due to MVAs, and further, sharing the results with politicians or the police. In contrast, driving may help elderly people to remain independent and mobile, and, therefore, revoking an elderly driver's license should be avoided. Similarly, making alternative transportation arrangements may also be essential in the case of a revoked driver's license. Environmental improvements for elderly drivers should be promoted in a balanced manner. In the super-aging society of Japan, it will be required to closely monito MVAs caused by elderly drivers.

### 4.1 Limitation

This study has several inherent limitations. First, this study did not grasp the all MVAs in Japan and perform a comprehensive analysis regarding the epidemiology of MVAs by elderly drivers, which might result in a selection bias. However, we consider that JTDB has the same degree of the representativeness of trauma databases in North America, Europe, and Oceania. Although there are approximately 3200 emergency medical institutions in Japan,^[28]^ the participating institutions include almost all of the certificated trauma educational ones and many tertiary care centers.^[10]^ Furthermore, as mentioned in the manuscript, JTDB is the only one prospective, nationwide, hospital-based trauma registry in Japan and supported by the large major associations,^[9,10]^ similar to trauma databases in North America, Europe, and Oceania. Secondly, this study focused only on elderly drivers themselves; as such, the prognosis of victims was not available in this registry. Third, this registry did not obtain the information about duplicated observations of the same individuals, so we could not exclude the duplicates. Finally, although we adjusted for potential confounders, there is the possibility of residual confounders.

## 5 Conclusion

In the super-aging society of Japan, the proportion of MVAs caused by elderly drivers increased year-by-year, and the mortality was highest in the those aged above 75 years. We should further investigate the trend between MVA incidence and outcomes as caused by elderly drivers, especially those of the old-old group, in order to decrease MVA mortality in Japan.

## Author contributions

**Conceptualization:** Tasuku Matsuyama, Tetsuhisa Kitamura, Nobunaga Okada, Kotaro Takebe, Makoto Watanabe, Yoshihiro Yamahata, Bon Ohta.

**Data curation:** Tasuku Matsuyama, Tetsuhisa Kitamura, Tomoya Hirose, Junya Sado.

**Formal analysis:** Tasuku Matsuyama, Kosuke Kiyohara, Junichi Izawa.

**Funding acquisition:** Tasuku Matsuyama.

**Investigation:** Tasuku Matsuyama, Kosuke Kiyohara.

**Methodology:** Takeyuki Kiguchi, Junichi Izawa, Yuki Miyamoto.

**Project administration:** Tetsuhisa Kitamura, Yusuke Katayama.

**Resources:** Tasuku Matsuyama, Junya Sado.

**Software:** Junya Sado.

**Supervision:** Kosuke Kiyohara, Bon Ohta.

**Writing – original draft:** Tasuku Matsuyama.

**Writing – review & editing:** Tetsuhisa Kitamura, Yusuke Katayama, Tomoya Hirose, Takeyuki Kiguchi, Junya Sado, Kosuke Kiyohara, Junichi Izawa, Nobunaga Okada, Kotaro Takebe, Makoto Watanabe, Yuki Miyamoto, Yoshihiro Yamahata, Bon Ohta.

Tasuku Matsuyama orcid: 0000-0003-4068-0306.

## Supplementary Material

Supplemental Digital Content
