# Supplementary material for: Motor vehicle accident mortality by elderly drivers in the super-aging era: A nationwide hospital-based registry in Japan
Source: Medicine (Baltimore). 2018 Sep 21;97(38):e12350. doi: 10.1097/MD.0000000000012350 (PMC6160118; doi:10.1097/MD.0000000000012350)
Supplement: Supplemental Digital Content [file medi-97-e12350-s001.docx]

| **Supplemental Table. Total number of institutions participating JTDB** | | | |  |  |  |  |  |  |  |  |  |
| --- | --- | --- | --- | --- | --- | --- | --- | --- | --- | --- | --- | --- |
|  | 2004 | 2005 | 2006 | 2007 | 2008 | 2009 | 2010 | 2011 | 2012 | 2013 | 2014 | 2015 |
| No. of participating institutions | 55 | 90 | 98 | 114 | 126 | 147 | 172 | 196 | 221 | 234 | 244 | 256 |
| JTDB indicates Japan Trauma Data Bank. |  |  |  |  |  |  |  |  |  |  |  |  |
